# Transparency of artificial intelligence/machine learning-enabled medical devices

**DOI:** 10.1038/s41746-023-00992-8

**Published:** 2024-01-26

**Authors:** Aubrey A. Shick, Christina M. Webber, Nooshin Kiarashi, Jessica P. Weinberg, Aneesh Deoras, Nicholas Petrick, Anindita Saha, Matthew C. Diamond

**Affiliations:** Center for Devices and Radiological Health, U.S. Food and Drug Administration, Silver Spring, MD USA

**Keywords:** Health policy, Medical ethics, Public health

## Introduction

Artificial intelligence/machine learning (AI/ML)-enabled medical devices (AI/ML devices, hereafter) offer exciting new opportunities to continue the advancement of healthcare. These innovative solutions can enable earlier disease detection, new insights into human physiology, improved and personalized diagnostics and therapeutics, and offer a unique ability to learn, adapt, and improve device performance as technologies and clinical contexts evolve or change^1^. AI/ML devices can harness the ever-growing amount of data available to actively learn from individuals using them during their lives, expanding the insights gained by decision makers, including healthcare providers, patients, caregivers, regulators, and payors^2^.

The United States Food and Drug Administration (FDA) is reviewing an increasing number of applications for AI/ML devices, with the number receiving FDA marketing authorization nearing seven hundred as of October 2023^3^. AI/ML devices have unique considerations during their development and use, including those for usability, equity of access^4^, management of performance bias^5^, the potential for continuous learning, and stakeholder (manufacturer, patient, caregiver, healthcare provider, etc.) accountability^6^. These considerations impact not only the responsible development and use of AI/ML devices but also the regulation of such devices^7^. FDA’s Center for Devices and Radiological Health (CDRH) recognizes these unique considerations and released an action plan for AI/ML devices in January 2021^8^. Among its numerous aims, this action plan highlights CDRH’s commitment to promoting transparency of AI/ML devices by fostering a patient-centered approach, while also collaborating with stakeholders on regulatory science efforts.

To advance efforts to promote the transparency of AI/ML devices further, CDRH hosted a virtual public workshop entitled “Transparency of Artificial Intelligence/Machine Learning-enabled Medical Devices” in October 2021^9^. Speakers, panelists, and attendees from many stakeholder groups, including patients, healthcare providers, researchers, industry members, regulators, and payors, participated. This well-attended workshop focused on identifying ways to achieve transparency for users (patient, caregiver, healthcare provider, etc.) of AI/ML devices, such as information-sharing mechanisms, and ways in which improved transparency might enhance the safety and effectiveness of these devices. This manuscript presents the takeaways from the workshop discussions on the meaning and role of transparency for stakeholders, as well as ways to promote transparency of all AI/ML-enabled software that meets the definition of a device^10^.

## The meaning and role of transparency

Since the definition of transparency may vary between stakeholders, CDRH proposed a working description for workshop participants. Workshop participants generally aligned with the idea presented: transparency is the degree to which appropriate information about a device—including its intended use, development, performance, and, when available, logic—is clearly communicated to stakeholders. Note, that the development and performance aspects include information on the data used in development and testing (e.g., sources, demographics). The concept encompasses both transparency related to device development, as well as transparency around a specific output or prediction from the AI/ML device that may impact how it is viewed or used. Some overarching considerations associated with the transparency of AI/ML devices that were discussed include information related to safety and effectiveness; testing for and mitigation of bias arising from differences in physical or social characteristics (including, but not limited to, race, ethnicity, gender, sex, age, disease, or medical condition severity); and monitoring of real-world performance and the impact real-world performance has on outcomes important to users. Building upon the working definition above, workshop participants articulated that clear communication is important to enable stakeholders’ understanding of the appropriate information about these characteristics throughout the total product life cycle. This can involve intentional planning of communication with users throughout the development process and subsequent deployment.

Workshop participants voiced that promoting and incorporating transparency is especially important for AI/ML devices as they are heavily data-driven, may incorporate algorithms exhibiting a degree of opacity^11^, and can potentially learn and change over time^12^. Transparency can support the proper use of an AI/ML device, allowing stakeholders to understand the role of the device within a clinical workflow (for example, knowing if the device is intended to inform, augment, or replace judgment of the user^13^) and make informed decisions.

Transparency also plays an important role in advancing health equity, which is a priority for CDRH^14^ and the Federal government^15^. Workshop participants stated that it is important for users to be aware of the population represented in the data used to develop and validate the AI/ML device, including associated physical or social characteristics. Furthermore, transparency on the device’s intended use, expected performance, demographic information of the data used in its development, and whether the data set is representative can help identify and manage bias that may impact a patient’s care. For example, a device trained and validated on older adults with diabetes may not work as well for pediatric patients with the same condition. Workshop participants also identified that improved transparency can also foster trust and confidence in the performance of AI/ML devices. Ultimately, transparency is a critical component to support the safe and effective use of AI/ML devices.

## Stakeholder perspectives

Workshop participants agreed on the need for transparency of AI/ML devices to more clearly communicate how a device works for an intended population, the presence and management of potential bias, and the role of the device in the clinical workflow. Multiple stakeholders expressed a need to establish a common framework and language around AI/ML devices in healthcare that can be leveraged to educate users, helping to empower them to make informed healthcare decisions.

Researchers at the workshop emphasized the importance of considering each stakeholders’ unique needs and tailoring information sharing to those needs, suggesting an opportunity for a human-centered design approach to transparency. This references the principles and practices of human factors engineering^16^ and human-centered design^17^. It focuses on a holistic approach to understanding, addressing, and involving users, their environments, and workflows to address the complex considerations associated with a given use case (like the use of AI/ML devices).

The subsequent sections expand on some workshop discussions, while Table 1 summarizes additional feedback stakeholders shared unique to their experiences. Taken together, the varied feedback provided by stakeholders reveals the opportunity for a human-centered approach to the transparency of AI/ML devices.

**Table 1:** Important transparency considerations expressed at the workshop.

Stakeholder	Transparency consideration
Patients	• Patient-centric labeling that includes information like training data demographics, technical requirements for use of device, etc. • Consistency in what information is shared • Variety of information delivery mediums (user interface, video, graphics, training, etc.) • Management of bias and patient-focused communication explaining these efforts • Appropriate notification of changes in device performance • Standard notation in electronic health records to indicate AI/ML device use, connecting events that could impact health (device recalls, device performance, etc.) • Trusted source(s) of device information
Healthcare providers	• Healthcare provider-specific labeling, specifically including intended use and performance in different populations • Consistency in what information is shared, including how a device can be used within the clinical workflow • Publicly available, detailed decision summary that includes information on physical or social characteristics of the clinical validation data • Standard testing before a device receives marketing authorization • Trusted source(s) of device information and user training resources • Notification to providers of device modification, malfunction, or performance changes • Real-world performance monitoring
Payors	• Assurance of performance across environments and populations • Real-world performance monitoring
Industry	• Appropriate level of regulatory oversight • Transparency aligned with proprietary needs • Additional guidance for device marketing authorization submissions • Post-market pathways to expand device claims

### Patients

For some patients and caregivers, a gap exists between their awareness or familiarity, if any, of AI/ML and their knowledge of how a specific AI/ML device could impact their health and healthcare, leading them to feel uncomfortable making decisions and deferring key shared decision making to their healthcare provider. Patients shared concerns that could be applicable in both situations where they used an AI/ML device and when their healthcare provider used an AI/ML device during their care. One key concern that emerged from patients at the workshop was whether AI/ML devices would inform or replace provider decisions, and how that could impact their care. Opportunities exist to empower patients by sharing educational resources, including questions to ask their healthcare provider (questions that could provide insight into a healthcare provider’s experience with and knowledge of an AI/ML device, questions about how a device performs for patients like themselves, questions about where they can find further information about a device, etc.). Building on the need for additional information, patients at the workshop also expressed their concern that a user’s technical literacy limits could further impact the quality of care they receive or the safety or effectiveness of the device they are using. Other transparency considerations identified as important to patients included data security and ownership, the cost of the device compared to the current standard of care, insurance coverage of the device, and the need for high-speed internet access or other technical infrastructure requirements.

### Healthcare providers

As with patients, some healthcare providers may be unfamiliar with how to use and effectively incorporate AI/ML devices into their clinical environment. At the workshop, providers expressed a desire to trust these devices at face value without needing an in-depth review to determine if they will work for their patients coming from various demographic groups and backgrounds that may not be captured in the data used to train or clinically validate the device. Providers voiced they may feel uncomfortable working with such devices as they currently find that information on AI/ML-device training, testing, and real-world performance may be difficult to understand or unavailable. Healthcare providers identified an opportunity to be more transparent in the delivery of this information not only in the data available and the media type in which it is communicated but also through who shares this information (device manufacturers, government agencies, professional societies, etc.). They expressed a need for transparency when communicating changes to the device and its performance and noted the importance of having a reliable mechanism to report device malfunction and performance drift to manufacturers.

### Payors

During the workshop, payor participants discussed that while the general trustworthiness of an AI/ML device can be demonstrated by its clinical use and validation, how the device specifically performs may vary by patient population, site, or environment of use. This can be particularly important to consider when an algorithm learns continuously instead of being “locked.” Given this potential for the AI/ML device to evolve, payors expressed concern with the coverage of “unlocked” or learning algorithms. Payor participants emphasized the importance of employing diversified datasets and discussed the possibility of monitoring the real-world performance of devices to ensure that they are performing as intended and improving patient outcomes.

### Industry

Industry members at the workshop shared their thoughts on a risk-based approach to transparency to maintain the least burdensome regulatory framework for AI/ML devices, while also mitigating potential proprietary risk that may arise with sharing information in an effort to be transparent. They expressed that their existing relationships with stakeholders are sufficient to communicate information about AI/ML devices to users through current device manuals, user training, and feedback processes. Workshop participants noted that the FDA is a trusted source of information for patients on manufacturers’ AI/ML devices and recommended manufacturers work with the FDA on transparent communications regarding these devices.

## Promoting transparency

In working towards the FDA’s mission to protect and promote public health, CDRH shares information on the safety and effectiveness of devices with the public through online databases (marketing authorization decision summaries, adverse event reports, recalls, etc.), letters to healthcare providers, safety communications, and guidance documents. The substantial amount of information available in these resources has the potential to better inform users on how a device might impact patients. However, workshop participants suggested that the delivery of this information, as well as the level of detail available, may not be sufficient to enhance stakeholder knowledge or their ability to make informed decisions. For these FDA communications and documents, one challenge is that much of the device information available on the CDRH website is developed by or geared toward manufacturers. Use of a complementary approach targeted to non-manufacturers to share information (e.g., graphics, plain language summaries) could allow the information to be more accessible for some stakeholders.

FDA also engages in regulatory science efforts^18^ to bring together stakeholders from across the ecosystem to identify and address cross-cutting areas of impact. Such areas may include best practices and standards for AI/ML devices, especially for the transparent communication of AI/ML device information and how considerations of a human-centered approach may affect such communication. Given the technical complexity of AI/ML devices and the unfamiliar nature of the concepts surrounding the development and use of AI/ML, such as training data or locked vs. continuous learning algorithms, expanded mediums and methods for information delivery are important to engender user trust. This could include using language appropriate for differing literacy, technical literacy, and health literacy levels, as well as accommodating those with different learning styles and delivery preferences. While AI/ML devices have already impacted numerous medical specialties, there are some nuances with the unique potential to impact patient care, such as change over time, bias, and intended role in clinical decisions. Workshop attendees identified that improving the transparency of AI/ML devices, especially concerning the communication of training, validation, and real-world performance, continues to be an area in need of further growth.

### Reporting summary

Further information on research design is available in the Nature Research Reporting Summary linked to this article.

### Supplementary information

updated Editorial Policy Checklist

Reporting Summary
